# 2. At the end of the road with a lifetime ahead: a challenging case of oligoarticular JIA in young adulthood

**DOI:** 10.1093/rap/rky031.001

**Published:** 2018-09-20

**Authors:** Susannah Brockbank, Julie Dawson

**Affiliations:** Rheumatology Department, St. Helens and Knowsley NHS Trust, St. Helens, UNITED KINGDOM

**Introduction:** We present a young patient with oligoarticular juvenile idiopathic arthritis (JIA) that has not responded to multiple conventional and biological disease modifying anti-rheumatic drugs (DMARDs) and highlight the biological, psychological and social barriers to his treatment.

**Case description:** The patient is a 20 year old man, who presented to our service at the age of 16 with a year-long history of monoarticular pain and swelling of the left knee. He had previously been fit and well and was a keen basketball player, an activity that he had had to stop doing due to his symptoms. Initial investigations revealed raised inflammatory markers and a normocytic anaemia but he is HLA B27, rheumatoid factor, anti-CCP and ANA negative. His symptoms responded poorly to intra-articular steroid treatment and the patient soon began to decline joint aspiration and injection due to needle phobia. He was initially treated with NSAIDs, followed by methotrexate (first orally, subsequently subcutaneously). Due to inadequate disease control, adalimumab was added to subcutaneous methotrexate. His joint disease has, however, failed to respond to these treatments and has progressed to involve his contralateral knee as well as his right shoulder and elbow. In view of his disease progression, his biologic treatment was switched to intravenous tocilizumab at four-weekly intervals. Though he had initially demonstrated a clinical response to tocilizumab and methotrexate in combination, he developed gastrointestinal symptoms that required discontinuation of methotrexate. Following this, his disease activity increased further and control could not be regained in spite of escalation of the tocilizumab frequency to two-weekly dosing intervals. He has therefore been switched to intravenous abatacept, though he has not yet demonstrated any clinical or biochemical response. Unfortunately, throughout this time he has had significant systemic disease manifestations with weight loss, ongoing anaemia and a CRP that is commonly in excess of 100mg/dl. In order to establish a degree of control of his disease, he has been treated with oral prednisolone but has now had protracted steroid therapy with doses in excess of 10mg daily to maintain his function. Despite ongoing steroid treatment, he has rapid radiographic progression and significant functional disability. Early in 2018, the patient developed acutely worsening right shoulder pain and a large effusion. In view of his immunosuppression and systemic symptoms, we were concerned that he had developed a septic arthritis. The patient declined joint aspiration, even under ultrasound guidance and thus we have been unable to exclude this diagnosis and have had to elect to continue his immunosuppression and monitor his progress clinically and radiologically. Repeated imaging of this joint has shown ongoing erosive disease with extensive synovial thickening and effusion. The paient's treatment has been complicated by inconsistent compliance with drug therapies and blood monitoring, partly due to his needle phobia but also due to a complex social situation. He is a lone parent and works full-time in a fast food restaurant - responsibilities that he finds difficult to balance with attending blood monitoring, infusion and follow-up appointments. This has posed a challenge to the department as his unpredictable attendance has meant that doses of high-cost biologic drugs have been wasted on a number of occasions. Special arrangements are now in place, in that the patient must attend the day unit prior to his infusion being ordered and wait on the day; however, the time that this takes presents a further barrier to his attendance. These arrangements highlight that the patient is not yet a fully independent, autonomous adult, in spite of being a parent himself. Furthermore, he does not seem to fully grasp the implications of his disease for his future.

**Discussion:** We present this case as the patient is now coming to the end of evidence-based, licensed and approved treatment pathways in JIA without adequate control of his disease. The next management options are uncertain, particularly in a general adult clinic with relatively small numbers of JIA patients and limited experience of this situation. This highlights the challenges faced when dealing with adults with persistent JIA, as the licensed treatment options are few and evidence-base sparse. In addition, this case highlights an important issue about managing young adults. A significant literature exists around the importance of transitional care in the adolescent population and the continuing unmet needs of this group. This literature eloquently describes the psychological and social barriers that JIA patients can face in young adulthood. This patient, however, presented directly to adult services and has therefore never experienced paediatric services or transition. Superficially, this would imply that he should be easier to manage as he does not have the social and psychological morbidity associated with a chronic disease in childhood. In spite of this, the challenges that he faces are demonstrably similar to post-transition young adults with JIA, indicating that planning and facilitating care for young adults goes beyond the provision of effective transition.

**Key Learning Points**: In order to manage the patient's care effectively in future, close collaboration between paediatric and adult rheumatology services is required. Experiences of managing JIA across the continuum is clearly necessary in order to plan long-term treatment. Alongside case discussions, there is clearly a role for collaboratively planning data collection and research to provide longitudinal data for outcomes of persistent, active JIA in adults. In addition, the provision of care for young adults in rheumatology needs to look beyond the management of transition in order to address the need of young people presenting to both paediatric and adult services. We wish to start an open conversation about experiences of managing adolescent patients, both in terms of planning drug treatment and the practical, psychological and social support that other centres offer in order to facilitate this treatment.

**Disclosure: S. Brockbank:** Other; SB has received funding from Pfizer and UCB for conference. **J. Dawson:** None.

